# Positive end-expiratory pressure optimisation during general anaesthesia in patients with obesity: a narrative review of respiratory and cardiovascular outcomes

**DOI:** 10.1016/j.bja.2025.09.009

**Published:** 2025-10-16

**Authors:** Christoph Boesing, Laura Schaefer, Patricia R.M. Rocco, Thomas Luecke, Joerg Krebs

**Affiliations:** 1 Department of Anaesthesiology and Critical Care Medicine, University Medical Centre Mannheim, Medical Faculty Mannheim of the University of Heidelberg, Mannheim, Germany; 2 Laboratory of Pulmonary Investigation, Carlos Chagas Filho Institute of Biophysics, Federal University of Rio de Janeiro, Centro de Ciências da Saúde, Rio de Janeiro, Brazil

**Keywords:** atelectasis, general anaesthesia, haemodynamics, lung-protective ventilation, obesity, perioperative care, positive end-expiratory pressure

## Abstract

Class III obesity is increasingly prevalent and presents unique perioperative challenges, particularly in the context of general anaesthesia and mechanical ventilation. The altered cardiopulmonary physiology in these patients increases susceptibility to alveolar collapse with impaired respiratory mechanics and gas exchange, significantly contributing to an increased risk of postoperative pulmonary complications. Positive end-expiratory pressure (PEEP) plays a pivotal role in lung-protective ventilation strategies but must be carefully titrated to balance its respiratory benefits against potential cardiovascular compromise. This narrative review explores the dual impact of PEEP on respiratory and cardiovascular outcomes during general anaesthesia in patients with obesity. We examine the obesity-related cardiopulmonary pathophysiology that influences the response to PEEP, including reduced lung compliance, increased pleural pressures, and altered venous return. Evidence from perioperative and critical care literature is synthesised to highlight the importance of PEEP in preventing atelectasis and improving oxygenation, while also considering its potential to impair cardiopulmonary function, particularly at higher levels. This review proposes a physiology-based framework and core recommendations to inform personalised PEEP management during general anaesthesia in patients with obesity, with the objective to optimise lung mechanics while preserving cardiovascular stability. We conclude that a nuanced, physiology-driven strategy to personalise PEEP, integrating respiratory mechanics, gas exchange, and cardiovascular parameters, can help optimise respiratory function and maintain cardiovascular stability in patients with obesity undergoing surgery. However, the clinical impact of such strategies needs to be confirmed in larger studies before they can guide evidence-based perioperative management in this high-risk population.

**Editor’s key points**

• Patients with obesity are susceptible to perioperative alveolar collapse and postoperative pulmonary complications. PEEP can improve respiratory mechanics, but the optimal method to set PEEP in this population is uncertain.
• This review presents a physiology-based framework with key recommendations to inform personalised PEEP management during general anaesthesia in patients with obesity, aiming to optimise pulmonary mechanics while maintaining cardiovascular stability.
• Although a personalised PEEP strategy can optimise pulmonary function and preserve cardiovascular stability, future studies are needed to evaluate its impact on patient-centred outcomes.

The global prevalence of class III obesity, defined by the World Health Organization as a BMI greater than 40 kg m^−2^, has increased significantly over the past decades^1^ and is estimated to affect approximately 25% of the adult population in developed countries by 2030.^2^ This escalating trend has led to a parallel increase in the number of individuals with obesity requiring surgical and interventional procedures under general anaesthesia.^3^ Class III obesity is frequently associated with a constellation of comorbidities, including obstructive sleep apnoea, metabolic syndrome, type 2 diabetes mellitus, and cardiovascular disease,^4^^,^^5^ which together contribute to increased risk of perioperative complications and mortality.^6^^,^^7^ Individuals with obesity are particularly vulnerable to the adverse physiological effects of general anaesthesia and positive pressure ventilation, that may exacerbate pre-existing cardiopulmonary dysfunction and significantly increase the risk of postoperative pulmonary complications (PPCs).^8^ Decreased functional residual capacity (FRC), elevated intra-abdominal pressure, and altered chest wall mechanics frequently lead to alveolar collapse and ventilation–perfusion mismatch during general anaesthesia. Notably, alveolar collapse plays a central role in the multifactorial pathogenesis of PPCs in patients with obesity.^8^^,^^9^ These complications—ranging from atelectasis to hypoxemia and respiratory failure—are a leading cause of prolonged hospitalisation and have been associated with up to a five-fold increase in perioperative mortality.^10^, ^11^, ^12^ Lung-protective ventilation strategies, that combine low tidal volumes (V_T_) with the judicious application of positive end-expiratory pressure (PEEP), are a cornerstone in mitigating these effects and minimising ventilator-induced lung injury.^13^

Although large randomised trials in patients with and without obesity have shown similar rates of PPCs when comparing low *vs* high or fixed PEEP strategies,^14^, ^15^, ^16^ recent meta-analyses support the use of personalised PEEP, tailored to individual respiratory mechanics and physiology, as a key component of an advanced lung-protective ventilation approach in patients or surgical conditions associated with a moderate to high PPC risk.^17^^,^^18^ When combined with alveolar recruitment manoeuvres (ARMs), personalised PEEP titration has consistently been associated with improved intraoperative respiratory mechanics, increased lung volumes, and enhanced gas exchange.^19^, ^20^, ^21^, ^22^, ^23^ Although these physiological improvements are often transient and tend to dissipate after extubation, current perioperative guidelines advocate for a patient-specific approach to PEEP management, particularly in patients with class III obesity, where lung mechanics are profoundly altered.^10^^,^^13^ Despite these advancements, the translation of temporary improved physiological variables into tangible, patient-centred outcomes remains a significant challenge. A deeper understanding of the complex interplay between obesity-related cardiopulmonary pathophysiology and the effects of positive pressure mechanical ventilation during general anaesthesia is essential to optimise perioperative PEEP strategies. In this context, the present review aims to critically examine the implications of PEEP management in patients with obesity undergoing general anaesthesia, with a particular focus on the cardiopulmonary consequences and potential for outcome improvement within a lung-protective framework.

## Pathophysiological consequences of obesity: implications for perioperative and respiratory care

**Fig 1:**
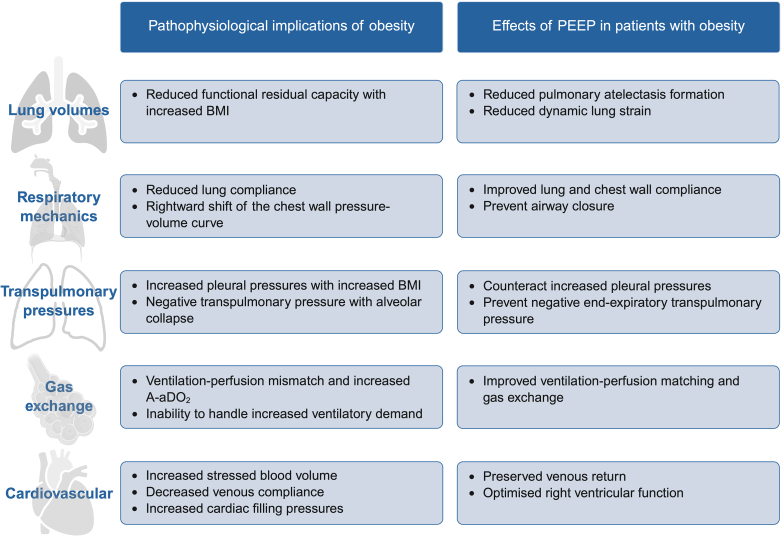
Pathophysiological implications of obesity and the role of PEEP in lung-protective ventilation strategies for patients with obesity. A-aDO_2_, alveolar-arterial oxygen difference; PEEP, positive end-expiratory pressure.

Class III obesity induces profound effects on pulmonary and cardiovascular function, directly influencing perioperative management strategies.^24^ This section outlines key pathophysiological changes in spontaneously breathing individuals with obesity, with emphasis on their implications for anaesthetic care and mechanical ventilation (Fig. 1).

### Lung volumes

A defining feature of obesity-related pulmonary physiology is the marked reduction in FRC,^25^^,^^26^ referring to the end-expiratory lung volume (EELV) under resting conditions. FRC represents the equilibrium between the inward elastic recoil of the lungs and the outward recoil of the chest wall.^27^ In individuals with obesity, excessive abdominal adiposity leads to a cephalad displacement of the diaphragm, whereas thoracic and pericardial fat deposition further accentuates intrathoracic pressure and reduces lung volumes.^26^^,^^28^^,^^29^

FRC decreases exponentially with increasing BMI, with reductions of approximately 5–15% for every 5 kg m^−2^ increase in BMI, depending on patient positioning.^25^^,^^30^^,^^31^ Central adiposity is particularly impactful, exacerbating FRC reduction beyond that observed with peripheral obesity.^32^ This decline is primarily attributable to a disproportionate decrease in expiratory reserve volume, whereas residual volume is typically preserved.^25^^,^^26^ Total lung capacity, determined by the compliance of the respiratory system and muscular function, is usually only mildly reduced even in class III obesity as a consequence of the preserved maximal inspiratory and expiratory pressures.^25^^,^^26^^,^^33^^,^^34^

### Respiratory mechanics

Obesity reduces the compliance of the respiratory system because of the combined effects on lung and chest wall mechanics. Reduced FRC is accompanied by decreased lung compliance, primarily caused by increased pulmonary blood volume, altered surfactant function, and the mechanical load of mediastinal and pericardial fat.^26^^,^^30^^,^^35^ These factors promote airway closure, particularly in dependent lung regions, thereby increasing the risk of atelectasis and impairing gas exchange.

In contrast, although thoracic adiposity shifts the pressure–volume curve of the chest wall to the right, it does not necessarily reduce chest wall compliance.^26^^,^^30^^,^^35^, ^36^, ^37^ Instead, the increased mass imposes an inspiratory threshold load—the airway pressure that must be exceeded before lung inflation can begin. Beyond this threshold, chest wall mechanics may remain relatively preserved, even in individuals with class III obesity.^26^^,^^38^

The work of breathing is consistently increased in individuals with obesity because of both increased airway resistance and the mechanical effort required to displace adipose tissue with each breath. Airway resistance is inversely related to lung volume, particularly FRC, making it inherently higher in these individuals.^39^ Additional contributors may include small airway remodelling caused by chronic low-grade inflammation, adipokine-mediated effects, and cyclical airway closure during tidal breathing.^40^, ^41^, ^42^ Although the forced expiratory volume in 1 s is generally only mildly decreased in most individuals with obesity,^43^ transitioning from an upright to a supine position further exacerbates airflow limitation and increases respiratory effort because of positional declines in lung volume.^44^

### Transpulmonary pressures

Individuals with obesity exhibit increased pleural pressures caused by the mechanical impact of thoracic and pericardial adipose tissue that exerts an inward force on the lungs and increases pressure within the pleural space.^26^^,^^28^^,^^29^ In addition, the substantial mass of abdominal fat leads to a cephalad displacement of the diaphragm, compounding this effect and further compressing the lung parenchyma.

Whereas pleural pressures in non-obese individuals typically range between −5 and +5 cm H_2_O,^45^ studies have shown that in individuals with obesity, pleural pressures can increase to approximately +5 cm H_2_O in the seated position and to more than 15 cm H_2_O in the supine position.^36^^,^^46^^,^^47^ During general anaesthesia, pleural pressures may increase further, reaching values as high as 30 cm H_2_O.^47^ The net result is a reduction in transpulmonary pressure (P_TP_), the difference between airway and pleural pressures that may fall below the threshold needed to maintain alveolar patency, thereby predisposing to alveolar collapse and contributing to reductions in FRC.

Moreover, a decrease in transmural airway pressure combined with reduced lung volumes can promote expiratory flow limitation during spontaneous breathing, especially in the presence of small airway closure.^48^ Supine positioning, which further increases pleural pressure relative to upright postures, can exacerbate these effects by reducing FRC and promoting dynamic airway compression.^34^^,^^41^^,^^49^^,^^50^

### Gas exchange

Gas exchange impairment in individuals with obesity may result from obesity-related comorbidities, such as obesity hypoventilation syndrome, or congestive heart failure, and from a decrease in FRC below the closing capacity during tidal breathing.^35^^,^^51^^,^^52^ This reduction in FRC promotes airway closure, predominantly in the dorsal (dependent) lung regions. Given that pulmonary perfusion increases from ventral to dorsal regions in the supine position, this regional mismatch between ventilation and perfusion causes right-to-left shunting and subsequent hypoxemia.^53^

The decrease in arterial partial pressure of oxygen (Pao_2_) and the increase in alveolar–arterial oxygen difference have been correlated with decreased lung volumes associated with increasing BMI.^54^ In individuals with a BMI of greater than 40 kg m^−2^, Pao_2_ is reduced by up to 20%, whereas alveolar–arterial oxygen difference may be up to four-fold higher compared with non-obese individuals.^55^ Central obesity and supine positioning further reduce FRC, thereby worsening gas exchange and contributing to hypoxaemia.^54^^,^^55^

Although individuals with obesity typically maintain normal arterial partial pressures of carbon dioxide at rest because of preserved respiratory muscle function, they may exhibit impaired ventilatory responses during exertion.^54^ This limitation is primarily attributable to the increased work of breathing and the diminished capacity to meet increased ventilatory demands.^55^

### Cardiovascular function

Individuals with obesity have an increased risk for cardiovascular disease attributable to the presence of metabolic syndrome that commonly includes arterial hypertension and ischaemic heart disease.^56^ The increased adipose tissue mass in obesity results in a greater circulatory demand, characterised by increased blood volume and cardiac output. These haemodynamic changes contribute to structural and functional cardiac alterations, such as ventricular hypertrophy, diastolic dysfunction, and atrial fibrillation. Furthermore, obesity, especially when associated with increased visceral adiposity, has been identified as a major risk factor for heart failure with preserved ejection fraction.^57^^,^^58^ In individuals with obesity, circulating blood volume is closely related to body mass. When combined with diastolic dysfunction, this expanded intravascular volume can increase cardiac filling pressures.^59^^,^^60^ Another contributing factor is increased pleural pressure, that can be higher than 15 cm H_2_O in spontaneously breathing individuals with obesity.^47^ As all intrathoracic structures, including the cardiac cavities and the adjacent great vessels, are affected by pleural pressure (‘a pressure chamber within a pressure chamber’), higher intraluminal pressures may be necessary to maintain adequate transmural pressure and effective chamber filling or vascular distension.

Venous return to the right heart, governed by the gradient between the mean systemic filling pressure—representing the upstream venous pressure—and right atrial pressure, may be compromised in this context. To maintain this gradient, venous compliance is reduced and stressed blood volume is increased in obesity, further exacerbating the cardiovascular load.^59^ In addition, pulmonary hypertension—either caused by obstructive sleep apnoea/obesity hypoventilation syndrome or secondary to increased left ventricular filling pressures—may further complicate the perioperative cardiovascular management of individuals with obesity.^61^

## Pulmonary effects of obesity during general anaesthesia: implications for positive end-expiratory pressure management

General anaesthesia with neuromuscular block and positive pressure mechanical ventilation is a well-established risk factor for perioperative pulmonary atelectasis.^62^^,^^63^ Atelectasis develops in up to 90% of patients undergoing general anaesthesia and impairs respiratory mechanics and gas exchange, thus promoting the development of other PPCs, such as pneumonia.^9^^,^^12^

In patients with obesity, the risk of significant pulmonary atelectasis is markedly increased resulting from three main contributing factors: increased pleural pressures, reduced alveolar pressure, and impaired surfactant function.^21^^,^^30^^,^^63^, ^64^, ^65^, ^66^, ^67^

After the induction of general anaesthesia and administration of neuromuscular block, the increased abdominal and thoracic adipose tissue exerts a cephalad pressure on the diaphragm, amplifying the force transmitted to the lung surface.^29^^,^^64^^,^^68^ Increased pleural pressures shift the pressure–volume relationship of the respiratory system to the right, promoting negative regional P_TP_ that drives alveolar collapse and airway closure.^47^^,^^69^ This risk increases in parallel with BMI because of the correlation with pleural pressures.^66^

In individuals with class III obesity, general anaesthesia can result in up to a 50% decrease in EELV, the lung volume present at end-expiration despite the application of PEEP. This reduction further exacerbates the already diminished FRC seen in spontaneously breathing individuals with obesity.^21^^,^^22^^,^^25^ During surgery, EELV in patients with obesity can fall to levels comparable with those seen in critically ill patients with severe acute respiratory distress syndrome.^29^^,^^70^^,^^71^ This profound lung volume loss can result in decreased respiratory system compliance, increased airway pressures, and severely impaired gas exchange, posing significant challenges for intraoperative ventilation management.^47^^,^^62^

Additional perioperative factors such as surgical positioning and pneumoperitoneum during laparoscopic procedures, mainly when associated with high insufflation pressures, can further exacerbate lung volume loss.^22^^,^^66^ The inflammatory response to surgery, combined with increased lung stress and strain from overdistension of the remaining aerated lung and high inspired oxygen concentrations, may further contribute to PPCs.^8^^,^^11^^,^^12^^,^^62^^,^^63^^,^^72^

Airway closure represents another key consequence of increased pleural pressures and excess adipose tissue in obesity. This phenomenon may be exacerbated by surgical positioning and pneumoperitoneum.^69^^,^^73^ When airway closure occurs, no gas reaches distal airways below a critical opening pressure, leading to misinterpretation of respiratory mechanics, cyclic opening and closing of small airways, and the development of denitrogenation atelectasis and surfactant dysfunction.^67^^,^^73^, ^74^, ^75^

Ultimately, pulmonary atelectasis and resultant ventilation–perfusion mismatch promote right-to-left shunting, impairing oxygenation and often requiring high inspired oxygen concentrations to avoid hypoxaemia. In the postoperative period, persistent alveolar collapse and reduced EELV place patients with obesity at heightened risk for oxygen desaturation and respiratory complications.^76^

## Role of positive end-expiratory pressure

As part of a lung-protective ventilation strategy, PEEP is used to reduce atelectasis and improve ventilation homogeneity by increasing P_TP_ (Fig. 1). When used appropriately, PEEP maintains EELV, enhances ventilation–perfusion matching, and improves respiratory mechanics and gas exchange.^19^, ^20^, ^21^, ^22^

Conceptually, PEEP should counteract the inward recoil forces acting on the lung that are amplified in obesity because of increased pleural pressures from both the thoracic adipose mass and reduced lung compliance.^77^ As a consequence of the respiratory system hysteresis, the pressure required to reopen collapsed alveoli (critical opening pressure) is substantially higher than the pressure at which they collapse during expiration (critical closing pressure).^78^ Therefore, an ARM is often necessary before PEEP titration to transiently achieve inspiratory P_TP_ levels above the critical opening pressure.^79^ In patients with obesity or during certain surgical conditions associated with increased pleural pressures (e.g. Trendelenburg position or pneumoperitoneum), airway pressures between 40 and 50 cm H_2_O may be required to achieve a sufficient inspiratory P_TP_ to reopen collapsed alveoli.^13^^,^^64^^,^^80^^,^^81^

After alveolar recruitment, PEEP must be maintained above the critical closing pressure to preserve alveolar stability, sustain EELV, and prevent derecruitment. Adequate PEEP also helps to minimise dynamic lung strain during inspiration, reflected by a lower respiratory system driving pressure, that represents the V_T_ normalised to aerated lung volume,^82^ and is closely associated with patient outcome.^19^^,^^83^^,^^84^ Conversely, excessive PEEP and P_TP_ can result in alveolar overdistension, increased lung stress, and strain and impairment of hemodynamics.^16^^,^^21^

In patients with obesity, pleural pressures increase in parallel with BMI and high levels of PEEP may be necessary to maintain non-negative end-expiratory P_TP_.^22^^,^^66^ However, pleural pressure distribution is regionally heterogeneous because of gravitational effects, mediastinal mass, and pericardial adiposity, producing a ventral-to-dorsal pleural pressure gradient. Consequently, the dependent lung regions are exposed to the highest pleural pressures.^44^

Oesophageal manometry using an oesophageal balloon catheter has been validated to approximate pleural pressures in the middle to the dependent lung^85^ and may be a valuable tool to personalise PEEP settings in patients with obesity.^86^^,^^87^ However, there is insufficient evidence regarding the regional distribution of pleural pressures in surgical patients with obesity.^44^ Although titrating PEEP to maintain a positive end-expiratory P_TP_ in the dependent lung can limit atelectasis in patients with class III obesity, this strategy may inadvertently increase the risk of overdistension in non-dependent lung areas, where pleural pressures are substantially lower.^22^^,^^44^

Numerous clinical studies have evaluated PEEP as part of lung-protective ventilation strategies during general anaesthesia and found improvements in physiological endpoints^19^, ^20^, ^21^, ^22^ and reduced incidence of PPCs in moderate- to high-risk surgical patients.^23^^,^^79^^,^^88^ However, three randomised trials in surgical patients without obesity reported no significant differences in outcomes, including the incidence of PPCs, when comparing fixed low with fixed high or personalised PEEP strategies.^14^^,^^15^^,^^89^ To date, the PROBESE study remains the only large-scale, multicentre randomised controlled trial comparing higher *vs* lower levels of PEEP in patients with obesity undergoing surgery.^16^ In this study, a fixed PEEP level of 12 cm H_2_O compared with 4 cm H_2_O was associated with improvements in respiratory mechanics and gas exchange, yet no significant reduction in PPC incidence was observed between the two groups.

These findings highlight two key considerations in interpreting the failure to translate improved physiological metrics into better clinical outcomes in surgical patients with obesity: (1) the appropriateness of the selected PEEP level and (2) the importance of personalised PEEP titration.

The PEEP level of 12 cm H_2_O used in the PROBESE study may have been insufficient to prevent loss of aerated lung volume due to pulmonary atelectasis formation, particularly in patients with class III obesity or in those undergoing surgery in conditions that increase pleural pressures (e.g. Trendelenburg position or laparoscopic surgery with pneumoperitoneum). Despite this, real-world intraoperative ventilation practices in patients with obesity tend to utilise even lower PEEP levels, with more than 75% of patients receiving ≤5 cm H_2_O.^10^

Several clinical studies in surgical patients with obesity demonstrated that PEEP levels greater than 12 cm H_2_O may be required to prevent extensive atelectasis formation, maintain EELV, and reduce respiratory variables associated with PPCs during general anaesthesia.^21^^,^^22^^,^^66^^,^^90^^,^^91^

In one study involving patients with a mean BMI of 41.9 kg m^−2^, a PEEP titration strategy guided by the highest dynamic respiratory system compliance resulted in a median PEEP of 15 cm H_2_O (interquartile range [IQR]: 13–17 cm H_2_O). Compared with a fixed PEEP of 8 cm H_2_O, the personalised PEEP approach was associated with improved intraoperative respiratory mechanics and gas exchange, and reduced postoperative atelectasis volume evaluated by computed tomography, which was statistically significant but of limited clinical relevance.^90^ Consistent with previous findings indicating that higher PEEP levels are required with increasing BMI to optimise lung function,^21^^,^^66^ the study also demonstrated a direct correlation between BMI and optimal PEEP.^90^

In a recent study including 40 patients with a mean BMI of 57.3 kg m^−2^, intraoperative PEEP titration guided by (1) the highest static respiratory system compliance and (2) an end-expiratory P_TP_ of 0 cm H_2_O yielded mean PEEP levels of 21.5 and 25.4 cm H_2_O, respectively, during laparoscopic procedures in the supine position.^22^ Although end-expiratory P_TP_ was slightly negative when PEEP was titrated to achieve the highest respiratory system compliance, this approach improved respiratory mechanics and gas exchange while preserving cardiac output. In contrast, targeting a positive end-expiratory P_TP_ may risk overdistension of non-dependent lung regions owing to the ventral-to-dorsal pleural pressure gradient. With regard to the negative end-expiratory P_TP_ in spontaneously breathing patients with class III obesity in the supine position,^47^^,^^50^ permitting a limited degree of atelectasis formation targeting a minimised inspiratory lung strain may represent a physiology-driven strategy to optimise lung mechanics and maintain cardiovascular stability.^22^ However, translating these short-term intraoperative physiological benefits into sustained postoperative improvements remains challenging. Additional interventions may be required in the postoperative period to maintain alveolar stability and support respiratory function in patients with obesity.

## Personalised positive end-expiratory pressure management

A personalised strategy to set PEEP, accounting for the heterogeneity in respiratory mechanics and intraoperative conditions, may be required to reduce the incidence of PPCs and improve clinical outcomes.^79^ The optimal PEEP level varies depending on BMI and surgical factors and a fixed PEEP setting is unlikely to be sufficient across all patients.^19^, ^20^, ^21^, ^22^^,^^66^^,^^91^

In a study involving patients with obesity under general anaesthesia with a mean BMI of 48.3 kg m^−2^, personalised PEEP titration guided by electrical impedance tomography (EIT) resulted in a mean PEEP of 18.5 cm H_2_O, with a range of 10–26 cm H_2_O.^21^ This strategy significantly increased EELV by 1.8 L and reduced driving pressure to 9.8 cm H_2_O, compared with 17.4 cm H_2_O when using a fixed PEEP of 5 cm H_2_O. Nevertheless, improvements in EELV, ventilation distribution and gas exchange disappeared within 2 to 6 h after extubation, suggesting the recurrence of atelectasis.

In addition to the BMI and the distribution of the adipose tissue mass, surgical factors have been shown to significantly affect respiratory mechanics. In this line, pneumoperitoneum during laparoscopic procedures increases intra-abdominal pressure, further reducing end-expiratory P_TP_, impairing respiratory system compliance, and promoting dorsobasal atelectasis formation.^92^^,^^93^ In such cases, increasing PEEP to offset the increased pleural pressure can restore P_TP_^94^ and help maintain respiratory mechanics, EELV, and gas exchange in patients with obesity.^22^ Conversely, intraoperative positioning in a more upright or beach-chair position reduces pleural pressure, enabling a lower PEEP requirement while preserving EELV and improving gas exchange.^22^

Dynamic intraoperative changes in pleural pressure, driven by surgical positioning, insufflation and abdominal contents, underscore the need for PEEP adjustment throughout the procedure. Tailoring PEEP to evolving intraoperative conditions is likely essential to maximise physiological benefits while minimising the risks of pulmonary overdistension and haemodynamic compromise associated with excessive PEEP.^20^^,^^22^^,^^66^ Given that the physiological improvements achieved with personalised PEEP often dissipate after extubation,^21^ further studies are needed to evaluate adjunctive postoperative interventions aimed at optimising respiratory function and preventing alveolar collapse. Such strategies may be key to translating intraoperative physiological gains into meaningful, patient-centred outcomes.

## Interplay between obesity-related cardiovascular pathophysiology and positive end-expiratory pressure during general anaesthesia

The application of PEEP during positive pressure mechanical ventilation can significantly affect cardiopulmonary function attributable to heart–lung interactions driven by changes in intrathoracic pressure. These effects are mediated by the PEEP-induced changes in lung volume, intrathoracic pressures, and the resulting consequences for cardiac preload, contractility, and afterload.^95^^,^^96^ Although the increased airway and intrathoracic pressures with PEEP are transmitted to all intrathoracic structures, the physiological consequences differ between the cardiac and pulmonary compartments. The heart and great vessels are primarily influenced by changes in pleural pressure, whereas the pulmonary circulation is affected by variations in P_TP_.

Under general anaesthesia, pleural pressures typically remain low (5–10 cm H_2_O) in non-obese individuals. However, in individuals with obesity, pleural pressure can be markedly increased, reaching levels as high as 25–30 cm H_2_O.^47^ This increase mitigates the cardiovascular impact of PEEP and must be carefully considered when titrating ventilatory support in this individuals.^97^^,^^98^

### Cardiac preload

PEEP modulates left ventricular preload, defined as left ventricular end-diastolic wall stress, by affecting systemic venous return, right ventricular output, and left ventricular filling.^96^ According to Guyton’s^99^ physiological model of venous return, an increase in right atrial pressure induced by PEEP theoretically decreases the pressure gradient for venous return, thus reducing right ventricular preload. However, in patients with obesity, this effect may be offset by increased pleural pressures, reduced venous compliance, and increased stressed blood volume.^59^^,^^60^ These compensatory mechanisms can maintain venous return despite higher levels of PEEP because of an increase in upstream venous pressures.^97^^,^^98^^,^^100^

### Pulmonary blood flow

PEEP affects right ventricular output by modifying pulmonary vascular resistance in response to changes in lung volume and hypoxic pulmonary vasoconstriction. Pulmonary vascular resistance is lowest near FRC, but in patients with obesity under general anaesthesia, significant atelectasis can increase right ventricular afterload.^101^ In addition, hypoxic pulmonary vasoconstriction may further increase pulmonary vascular resistance and impede right ventricular ejection.^102^^,^^103^ Adequate PEEP can promote alveolar recruitment and enhance aeration of lung parenchyma,^22^ thereby reducing pulmonary artery pressure and right ventricular afterload.^98^ In contrast, pulmonary overdistension caused by excessive P_TP_ may increase pulmonary vascular resistance and right ventricular afterload. Another mechanism that may impede right ventricular ejection is a large inspiratory strain,^100^^,^^104^ clinically observed as increased driving pressures because of extensive alveolar collapse.^22^ Therefore, careful balancing of PEEP to optimise recruitment without overdistension and avoidance of large intrathoracic pressure fluctuations is essential for preserving right ventricular performance and, through ventricular interdependence, maintaining left ventricular preload.^98^^,^^105^, ^106^, ^107^

### Cardiac contractility and afterload

Left ventricular stroke volume is determined by preload, intrinsic myocardial contractility, and afterload. Although PEEP may influence preload and diastolic function in non-obese individuals,^108^ it has not been shown to significantly affect left ventricular contractility.^109^^,^^110^ However, PEEP can reduce left ventricular afterload and stroke work by reducing left ventricular transmural pressure (difference between intracavitary and pleural pressure), through an increase in pleural pressure. This effect can enhance left ventricular ejection fraction.^111^

In patients with obesity emerging from general anaesthesia, the combination of pulmonary atelectasis,^76^ increased work of breathing,^112^ and potential upper airway obstruction can result in marked decreases in intrathoracic pressure. This can increase both left ventricular afterload and venous return, with the potential to cause pulmonary oedema and extubation failure.^113^^,^^114^

## Possible adverse haemodynamic effects of positive end-expiratory pressure during general anaesthesia

PEEP in combination with ARMs may adversely affect haemodynamic and increase the need for fluid administration and cardioactive drugs that could impact patient outcomes.^16^^,^^21^^,^^115^ The predominant cause for PEEP- and ARM-induced haemodynamic impairment during general anaesthesia in recent studies has been discussed as arising from an increase in intrathoracic (pleural) pressure that decreases the gradient for venous return, particularly in the presence of hypovolaemia^14^ and may cause hypotension or bradycardia.^16^^,^^21^ However, in patients with obesity, increased pleural pressures protect the cardiovascular system against higher airway pressures associated with PEEP, and preventing extensive atelectasis formation and hypoxaemia may even improve right ventricular ejection.^97^^,^^98^

In contrast to the clinical trials comparing fixed low *vs* high PEEP in surgical patients without obesity, recent studies evaluating personalised PEEP strategies in patients with obesity demonstrated preserved haemodynamic without differences in cardiac output, oxygen delivery, or vasopressor requirements.^22^^,^^90^^,^^116^ The protective effect of high pleural pressures were highlighted in a recent study using oesophageal pressures as a surrogate for pleural pressure to personalise PEEP according to end-expiratory P_TP_.^22^ In this study, cardiac output remained stable even with PEEP levels above 20 cm H_2_O, as expiratory oesophageal pressures were in the range of 20–30 cm H_2_O. This confirms the findings of previous trials in critically ill patients with obesity with and without acute respiratory distress syndrome, in which a high PEEP to avoid negative end-expiratory P_TP_ improved respiratory mechanics without causing haemodynamic impairment.^87^^,^^97^^,^^117^

Maintaining EELV by applying adequate PEEP may be protective for the lungs and the heart because large swings in intrathoracic pressure and cyclic alveolar overdistension impair right ventricular ejection and pulmonary blood flow.^98^^,^^100^^,^^101^ This should be weighed against the possible reduction in cardiac preload associated with higher PEEP, especially in patients with obesity and severe cardiovascular disease or volume depletion.

The cardiovascular effects of ARMs, characterised by transient increases in airway and intrathoracic pressures above PEEP, can be clinically significant and are associated with bradycardia, hypotension, and the requirement for vasoactive agents, even in patients with obesity.^13^^,^^16^^,^^115^ In the PROBESE trial, an intraoperative ventilation strategy with a fixed PEEP of 12 cm H_2_O combined with repetitive ARMs resulted in a higher incidence of hypotension compared with a strategy using a fixed PEEP of 4 cm H_2_O without ARMs.^16^ Notably, the PROBESE trial did not differentiate between the haemodynamic effects occurring during ARMs and those occurring outside of them. However, a secondary analysis including all PROBESE patients from a single centre, along with data from a local physiological study, found no significant differences in haemodynamic parameters or vasopressor requirements outside of ARMs across groups receiving a fixed PEEP of 4 cm H_2_O, a fixed PEEP of 12 cm H_2_O, or individualised PEEP (median 18 cm H_2_O).^91^

These findings suggest that in surgical patients with class III obesity, elevated pleural pressures may mitigate the risk of clinically relevant haemodynamic impairment associated with higher PEEP levels. In contrast, ARMs—particularly in patients with underlying cardiovascular disease, hypovolaemia, or circulatory instability—can precipitate significant haemodynamic compromise. Furthermore, excessive PEEP or P_TP_ leading to alveolar overdistension may impair venous return and increase right ventricular afterload, exacerbating cardiovascular strain.

Given the variability in pleural pressures based on BMI, intraoperative conditions, and cardiopulmonary reserve, a holistic, personalised approach to PEEP titration is essential. This strategy should aim to balance the potential respiratory benefits with the risk of haemodynamic impairment.

## Positive end-expiratory pressure management in clinical practice

PEEP is recommended as part of a lung-protective ventilation strategy with the aim to minimise atelectasis and preserve EELV, thereby promoting homogeneous ventilation distribution, decreasing inspiratory lung strain, and improving gas exchange.^13^ Given the considerable heterogeneity among patients with obesity and intraoperative conditions, a one-size-fits-all approach might be inadequate; instead, a personalised strategy to optimise PEEP might be prudent (Table 1).^13^^,^^79^ Recent studies using physiologic variables such as P_TP_ and bedside imaging techniques such as EIT have demonstrated that PEEP levels exceeding 15 cm H_2_O may be necessary in patients with obesity to optimise respiratory mechanics and improve gas exchange.^21^^,^^22^^,^^90^^,^^91^^,^^118^

**Table 1:** Core recommendations for PEEP optimisation during general anaesthesia in patients with obesity. PEEP, positive end-expiratory pressure.

Recommendation	Description
Anticipate obesity-related cardiopulmonary pathophysiology	Understand the impact of obesity on respiratory physiology during general anaesthesia, including reduced functional residual capacity, altered chest wall mechanics, elevated pleural pressures, and ventilation–perfusion mismatch.
Identify risk factors for intraoperative atelectasis	Recognise clinical and surgical factors predisposing to alveolar collapse, such as high BMI, central adiposity, pneumoperitoneum, and Trendelenburg position.
Personalise PEEP after alveolar recruitment	Perform an alveolar recruitment manoeuvre followed by personalised PEEP titration to stabilise recruited alveoli, minimise end-expiratory collapse, and reduce tidal overdistension.
Evaluate the effectiveness of PEEP and monitor for possible adverse effects	Continuously assess the impact of PEEP on respiratory mechanics—such as driving pressure and respiratory system compliance—and on gas exchange. Simultaneously, monitor for potential adverse cardiovascular effects. In patients with haemodynamic instability, efforts should be made to minimise the cardiovascular impact of mechanical ventilation by avoiding repetitive alveolar recruitment manoeuvres and by carefully balancing the goals of lung protection with the need to preserve haemodynamic stability.

A practical and widely applicable approach in clinical settings is to titrate PEEP based on the highest static respiratory system compliance.^79^ This method, based on a strong physiological rationale, targets minimisation of driving pressure, a surrogate for inspiratory lung strain, by balancing alveolar recruitment and overdistension.^19^^,^^23^^,^^83^^,^^84^ It is feasible to utilise this strategy with most modern anaesthesia ventilators and there is strong concordance with advanced bedside imaging techniques.^119^ Although performing a static measurement with zero gas flow by using an inspiratory pause or hold manoeuvre to compute the static respiratory system compliance is the preferred method, the dynamic respiratory system compliance using the peak airway pressure can be used as a surrogate.^90^^,^^118^

**Fig 2:**
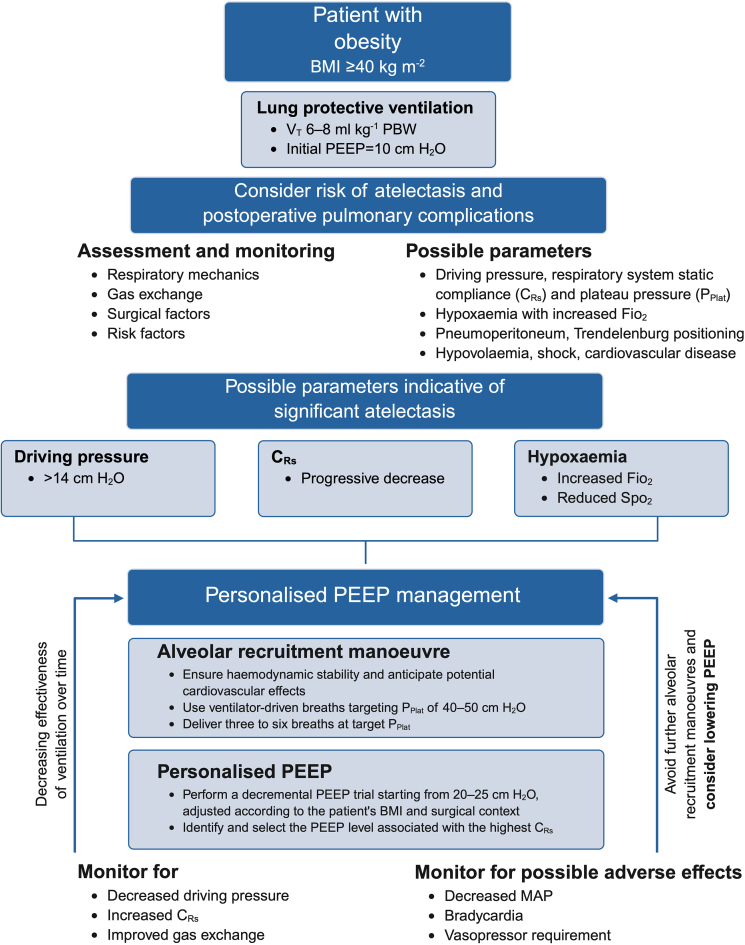
Algorithm to identify possible clinical parameters indicative of significant pulmonary atelectasis and to personalise PEEP management in patients with obesity. C_R__s_, respiratory system static compliance; Fio_2_, fractional of inspired oxygen; MAP, mean arterial pressure; PBW, predicted body weight; PEEP, positive end-expiratory pressure; P_Plat_, respiratory system plateau pressure; Spo_2_, peripheral oxygen saturation; V_T_, tidal volume.

A personalised approach to set PEEP necessitates a comprehensive assessment of the risk for pulmonary atelectasis formation and other PPCs. This includes evaluation and monitoring of respiratory mechanics (airway pressures including driving pressure and respiratory system compliance), gas exchange, and surgical factors (positioning and pneumoperitoneum insufflation pressure), with the latter also contributing to changes in chest wall compliance (Fig. 2).^94^^,^^120^ Furthermore, a careful risk–benefit assessment of cardiopulmonary function should be performed with the aim of detecting risk conditions associated with haemodynamic impairment during ARMs and PEEP titration, such as cardiovascular disease, hypovolaemia, and circulatory shock.

After anaesthesia induction or if significant atelectasis formation is suggested by driving pressure that is high, increasing, or both; progressively decreasing respiratory system compliance; and worsening gas exchange with hypoxaemia, a systematic approach with the aim of recruiting lung parenchyma and increasing EELV by increasing P_TP_ is indicated. After an ARM,^13^ a decremental PEEP trial can be conducted in a volume-controlled ventilation mode, beginning with a PEEP of 20–25 cm H_2_O, adjusted according to the patient's BMI and surgical context. PEEP is then decreased in 2 cm H_2_O steps every 10 breaths, while continuously monitoring static respiratory system compliance. The trial continues until no further improvement in compliance is observed. If comparable compliance values are achieved at multiple PEEP levels, the lowest PEEP that maximises static respiratory system compliance should be selected for ongoing ventilation.^22^ The effectiveness of the ARM and the set PEEP should be evaluated immediately using respiratory mechanics and gas exchange, with particular attention given to potential adverse effects (Fig. 2). Given the dynamic nature of intraoperative conditions, ongoing reassessment of PEEP is essential to maintain its benefits while minimising potential harms.

## Conclusions

In patients with class III obesity, development of perioperative alveolar collapse and the associated impairment in respiratory mechanics and gas exchange are key drivers of PPCs and increased perioperative mortality. Optimising PEEP management requires a comprehensive and personalised assessment of atelectasis risk in the context of cardiopulmonary function and surgical factors to balance respiratory benefits with potential cardiovascular effects.

A personalised PEEP strategy, applied within a lung-protective ventilation approach, may reduce alveolar collapse, optimise respiratory system compliance, and preserve cardiovascular stability in patients with obesity undergoing surgery; however, its clinical impact requires confirmation in larger studies to guide evidence-based perioperative management in this high-risk population.

## Authors’ contributions

Study design and conception: all authors

Literature search and selection: CB, LS, JK

Data extraction and synthesis: CB, LS

Writing: CB, JK

Critical revision: CB, PRMR, TL, JK

Approved the final version of the manuscript: all authors

## Funding

Brazilian Council for Scientific and Technological Development and Carlos Chagas Filho, Rio de Janeiro State Foundation (Fundação de Amparo à Pesquisa do Estado do Rio de Janeiro) (PRMR) ; Bundesministerium für Bildung und Forschung (JK); Hamilton Medical and Ebenbuild (JK).

## Declaration of interest

The authors declare that they have no conflicts of interest.
